# Editorial: Interfascial Plane Blocks

**DOI:** 10.3389/fmed.2022.962487

**Published:** 2022-07-29

**Authors:** Alessandro De Cassai, Fabio Costa

**Affiliations:** ^1^Department of Medicine, Anaesthesia and Intensive Care Unit, University Hospital of Padua, Padua, Italy; ^2^Unit of Anaesthesia, Intensive Care and Pain Management, Department of Medicine, Università Campus Bio-Medico di Roma, Rome, Italy

**Keywords:** regional anesthesia, interfascial plane block, education, safety, editorial

After the first publication on the TAP block by Rafi (1), we witnessed an exponential increase in interest in interfascial plane blocks (IPBs). IPBs are a group of regional anesthesia techniques aiming to deposit an injectate, typically local anesthetic, in a fascial plane such that it spreads within a potential space to affect one or more neural targets. Sometimes neural targets in the fascial plane are too small and therefore difficult or impossible to visualize, for instance the case of TAP blocks, interpectoral blocks, or PENG blocks. Sometimes those targets are great enough to be visualized, but targeting the fascial plane, instead of the nerve itself, increases the longitudinal spread and reduces the incidence of nerve injuries, for instance, the fascia iliaca blocks for the femoral nerve together with the lateral cutaneous femoral nerve, the subpectineus technique for the common obturator nerve or the parasacral ischial plane block for the sciatic nerve.

The introduction of ultrasound guidance largely contributed to this growth in interest for IPB. Ultrasound added safety and effectiveness as further factors that facilitated this expansion. When the proper skills for ultrasound machine handling are achieved, the appropriate knowledge of the relevant anatomy allows the anesthesiologist to tailor the analgesia over the surgical requirements, accomplishing a complete analgesia field by adding pieces, one over another, like a jigsaw puzzle (2). As an example, adding an interpectoral block with a parasternal block and a pecto-serratus block, allows completion of the field of analgesia for modified radical mastectomies, as well as the combination of a pecto-serratus block with a supraclavicular block of the brachial plexus, allows to obtain surgical analgesia for a trans-axillary correction of the thoracic outlet syndrome (3).

With the proper skills, the safety and ease of IPBs permits non-anesthesiologist clinicians to effectively perform these procedures in breast surgery settings (4).

This growth leads to two main issues: on the one hand, the introduction of these new blocks overwhelmed scientific journals with similar, if not outright identical, block procedures published under different names creating confusion among anesthesiologists; on the other hand, the introduction of IPBs introduced a set of novel skills and knowledge that anesthesiologists should attain in order to successfully apply them in the clinical practice (4, 5).

The first issue has been discussed in a conjunct ESRA/ASRA consensus reaching a list of standardized blocks (6); however, a recent review showed that the novel proposed nomenclature has been only partially adopted by the scientific community (7).

The second issue is the delay in the implementation of IPB training. Improper block selection associated with inadequate needle handling and often with an inappropriate needle target may cause (8, 9), serious complications such as pneumothorax and motor block as reported (10). The lack of proper training programs is a void to be filled in the very next future.

A brilliant example of an educational program for IPBs has been presented in the paper by Torrano et al. showing how educational courses and programs dedicated to IPBs are able to reduce the procedure time and the number of needle insertions to obtain a successful block and at the same time increasing the operators' confidence.

As previously stated, IPBs are a wide family of regional techniques with a wide range of indications. However, only in the last few years, an increasing number of randomized controlled trials have been performed permitting an overall analysis of their true effectiveness and applicability in different clinical scenarios.

For this reason, we are delighted that in this special issue of Frontiers we were able to collect both randomized controlled trials (Xu et al.) and meta-analysis (Viderman et al.a; Viderman et al.b), showing that both the interest and the evidence for this field of regional anesthesia is growing.

The published articles in this issue represent a small but important contribution to the IPB field. However, we have discovered only a few pieces of the IPB puzzle, and we look forward to being able to examine the full picture in the next few years.

## Author Contributions

Both authors wrote, read, and approved the final draft of the manuscript.

## Conflict of Interest

The authors declare that the research was conducted in the absence of any commercial or financial relationships that could be construed as a potential conflict of interest.

## Publisher's Note

All claims expressed in this article are solely those of the authors and do not necessarily represent those of their affiliated organizations, or those of the publisher, the editors and the reviewers. Any product that may be evaluated in this article, or claim that may be made by its manufacturer, is not guaranteed or endorsed by the publisher.
